# Reducing False-Positive Results in Newborn Screening Using Machine Learning

**DOI:** 10.3390/ijns6010016

**Published:** 2020-03-03

**Authors:** Gang Peng, Yishuo Tang, Tina M. Cowan, Gregory M. Enns, Hongyu Zhao, Curt Scharfe

**Affiliations:** 1 Department of Genetics, Yale University School of Medicine, New Haven, CT 06520, USA; gang.peng@yale.edu (G.P.); yishuo.tang@yale.edu (Y.T.); hongyu.zhao@yale.edu (H.Z.); 2 Department of Biostatistics, Yale University School of Public Health, New Haven, CT 06520, USA; 3 Department of Pathology, Stanford University School of Medicine, Stanford, CA 94304, USA; tcowan@stanfordhealthcare.org; 4 Department of Pediatrics, Stanford University School of Medicine, Stanford, CA 94304, USA; greg.enns@stanford.edu

**Keywords:** newborn screening, inborn metabolic disorders, tandem mass spectrometry, false positive, second-tier testing, machine learning, Random Forest

## Abstract

Newborn screening (NBS) for inborn metabolic disorders is a highly successful public health program that by design is accompanied by false-positive results. Here we trained a Random Forest machine learning classifier on screening data to improve prediction of true and false positives. Data included 39 metabolic analytes detected by tandem mass spectrometry and clinical variables such as gestational age and birth weight. Analytical performance was evaluated for a cohort of 2777 screen positives reported by the California NBS program, which consisted of 235 confirmed cases and 2542 false positives for one of four disorders: glutaric acidemia type 1 (GA-1), methylmalonic acidemia (MMA), ornithine transcarbamylase deficiency (OTCD), and very long-chain acyl-CoA dehydrogenase deficiency (VLCADD). Without changing the sensitivity to detect these disorders in screening, Random Forest-based analysis of all metabolites reduced the number of false positives for GA-1 by 89%, for MMA by 45%, for OTCD by 98%, and for VLCADD by 2%. All primary disease markers and previously reported analytes such as methionine for MMA and OTCD were among the top-ranked analytes. Random Forest’s ability to classify GA-1 false positives was found similar to results obtained using Clinical Laboratory Integrated Reports (CLIR). We developed an online Random Forest tool for interpretive analysis of increasingly complex data from newborn screening.

## 1. Introduction

Newborn screening (NBS) using tandem mass spectrometry (MS/MS) has transformed our ability to identify and provide early, lifesaving treatment to infants with hereditary metabolic diseases. Because screening is designed to identify affected infants at high sensitivity, it is accompanied by frequent false-positive results [1]. Additional biochemical and DNA testing of all screen-positive cases is performed to confirm (true positive) or reject (false positive) the primary screening result and to reach a final diagnosis. In some cases, this two-tier strategy can lead to iterative testing rounds and diagnostic delays, placing undue burden on the healthcare system including physicians and clinical laboratories, and on the patients and their families.

At present, only one or a few metabolic analytes or ratios from MS/MS screening panels are used to identify infants with a metabolic disorder. For example, screen-positive cases for methylmalonic acidemia (MMA) are identified using specific cutoff values for propionylcarnitine (C3) and its ratio with acetylcarnitine (C2), two of the 39 analytes measured in MS/MS screening. As an alternative approach to analyte cutoffs, Clinical Laboratory Integrated Reports (CLIR, formerly R4S) postanalytical testing employs a large database of dynamic reference ranges for disease-related analytes and many additional informative analyte ratios in order to improve separation of true- and false-positive cases [2,3,4,5]. The ranges and overlap of analyte values between patient and control groups can be adjusted in CLIR for multiple continuous and clinical variables (e.g., birth weight, sex, age at blood collection), which have been shown to significantly reduce false-positive results [6].

Machine learning is an emerging strategy for the classification of metabolic disorders in newborns [7,8]. In particular, Random Forest (RF) or Random Decision Forests [9,10] are powerful tree-based methods for supervised machine learning with numerous applications in high-throughput genomic [11] and metabolomic data analysis [12,13,14]. We recently showed that analysis of all 39 MS/MS analytes in the California NBS panel using RF was able to improve the separation of true- and false-positive cases [15]. We compared results from our RF analysis to results obtained from CLIR for the same cohort of MMA screen positives. This comparison showed that the prediction of MMA false positives was significantly improved by utilizing the entire set of MS/MS analytes measured at birth. Here we adapted our RF approach developed for methylmalonic acidemia (MMA) [15] to the study of additional metabolic disorders to improve the diagnosis of glutaric acidemia type 1 (GA-1) and very long-chain acyl-CoA dehydrogenase deficiency (VLCADD); and facilitate detection of ornithine transcarbamylase deficiency (OTCD) that is not currently on the Recommended Universal Screening Panel (RUSP) [16]. The performance and stability of the RF model was evaluated using NBS data from screen-positive infants for these disorders reported by the California NBS program. Based on these findings, we developed open-source web-based software (https://rusptools.shinyapps.io/RandomForest) that incorporates our RF model for the analysis and interpretation of newborn screening data. The new RF tool could be used to identify false-positive results in conjunction with CLIR tools and established second-tier confirmatory testing using biochemical and DNA analysis of all screen-positive cases.

## 2. Materials and Methods

### 2.1. Data Summary

This study was approved by the Institutional Review Boards at Yale University (protocol ID 1505015917, 10 May 2019), Stanford University (protocol ID 30618, 25 February 2019) and the State of California Committee for the Protection of Human Subjects (protocol ID 13-05-1236, 7 June 2019). We analyzed newborn screening data from a cohort of 2777 infants, consisting of 235 cases with confirmed glutaric acidemia type 1 (GA-1), methylmalonic acidemia (MMA), ornithine transcarbamylase deficiency (OTCD), or very long-chain acyl-CoA dehydrogenase deficiency (VLCADD), and 2542 false positives for one of these disorders (Table 1). A number of confirmed positive cases had metabolic marker concentrations below the established cutoff values and thus were not technically screen positive for the respective disease. Positive predictive value (PPV) was calculated after removing these cases, which included 5 of the 48 GA-1 cases and 4 of the 103 MMA cases. OTCD is detected through decreased citrulline levels and 6 of the 24 confirmed positive OTCD cases had levels above the established cutoff. All babies had newborn screening performed through the California NBS program between 2005 and 2015, except for OTCD, which was performed between 2010 to 2015. Data included 39 analytes (free carnitine, 26 acylcarnitines, and 12 amino acids), as well as gestational age (GA, in days), birth weight (BW, in grams), sex (male, female or unknown), race/ethnicity status that was self-reported by the parents, age at blood collection (AaC, in hours), and total parenteral nutrition (TPN, yes or no) status (Table 2).

### 2.2. NBS Metabolic Data Analysis Using Random Forest

**Figure 1:**
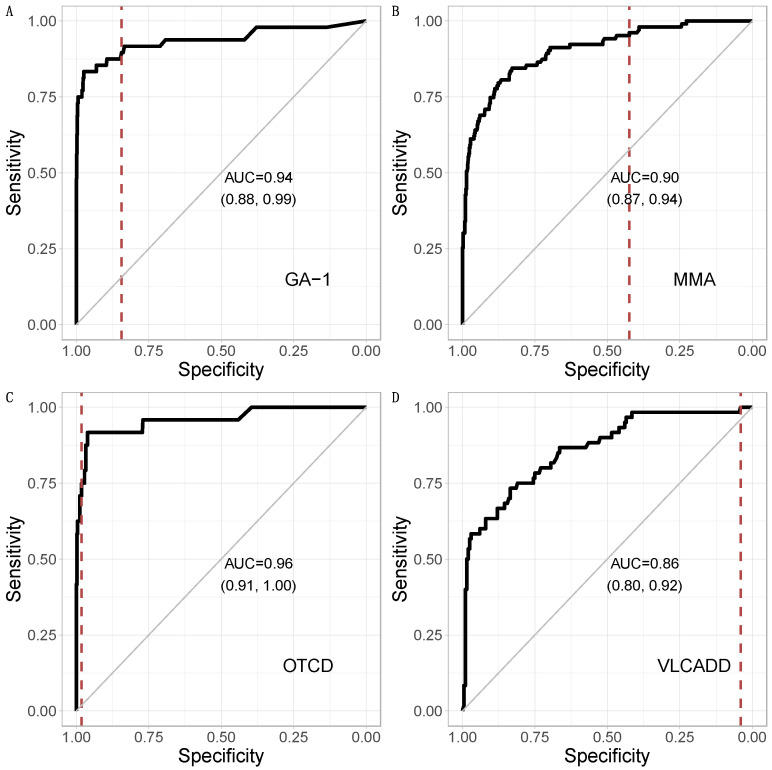
Analysis of newborn metabolic profiles with Random Forest (RF). Receiver operating characteristic (ROC) curve analysis for infants with and without a confirmed diagnosis using RF analysis of 39 MS/MS analytes. Without altering the sensitivity of primary newborn screening (NBS) for each of the four disorders, RF reduced the number of false-positive cases (vertical dotted line) by 89% for GA-1 (**A**), 45% for MMA (**B**), 98% for OTCD (**C**) and by 2% for VLCADD (**D**). For each disease, the number in parenthesis shows the 95% confidence interval of the area under the ROC curve (AUC).

Random Forest [10] was used to evaluate information from all 39 metabolic analytes and the six clinical variables (GA, BW, sex, race/ethnicity, AaC, TPN) in the 2777 screen-positive cases. In this study, analyte ratios were not included in the RF model due to: 1) Difficulty selecting ratios from the large number of possible ratio for 39 analytes, 2) Ability of RF decision trees to capture nonlinear relationships between analyte ratios (e.g., change of C3 in relation to C2) and 3) Possibility that adding ratios could create a bias in the ranking of analytes using mean decrease in accuracy (MDA). One-hot-encoding was used to convert the following three categorical variables into a form that could be used by the machine learning algorithm: sex (male, female, or sex-NA), race/ethnicity (Asian, Black, Hispanic, White, or Other/Unknown) and TPN (TPN–Yes, TPN–No, or TPN–NA). Leave-one-out cross-validation (LOOCV) was used to estimate the reliability of RF to correctly predict true- and false-positive cases. For each disease, the RF model was trained on all screen-positive cases for that disease except for one blinded case for which a prediction was made. This process was repeated for all screen-positive cases for each disease. For example, 604 MMA screen positives were combined for training, while 1 MMA screen-positive case was blinded and used for testing. This process was repeated until all 605 MMA cases were classified by RF. Only RF assignments from testing cases (and not from training) were used for final outcome prediction. This prediction was based on counting the “vote” from each RF decision tree with a binary classification of only two possible outcomes: a screen positive can either be true positive or false positive for the disorder. The number of decision trees was set to 1000 in each RF model [17]. The fraction of decision trees that voted for a case as true positive among all 1000 decision trees was defined as the RF score in this study. In result of the LOOCV, one RF score was assigned to each of the 605 MMA screen positive that ranged from 0 to 1. This RF score was used to plot the receiver operating characteristic (ROC) curve (Figure 1), and to calculate the area under the curve (AUC). The ROC curve shows the correlation between sensitivity and specificity at different cutoffs of the RF score. AUC indicates the performance of the model with range between 0.5 and 1. AUC of value 1 indicates a perfect model for separating true and false positives, while 0.5 has no class separation capacity. A high RF score indicates a high probability of a case being a true positive, while a low RF score indicates a high probability of a false-positive NBS result. There is a direct correlation between the RF score and screening sensitivity and specificity.

### 2.3. Validation of the Random Forest Model

**Figure 2:**
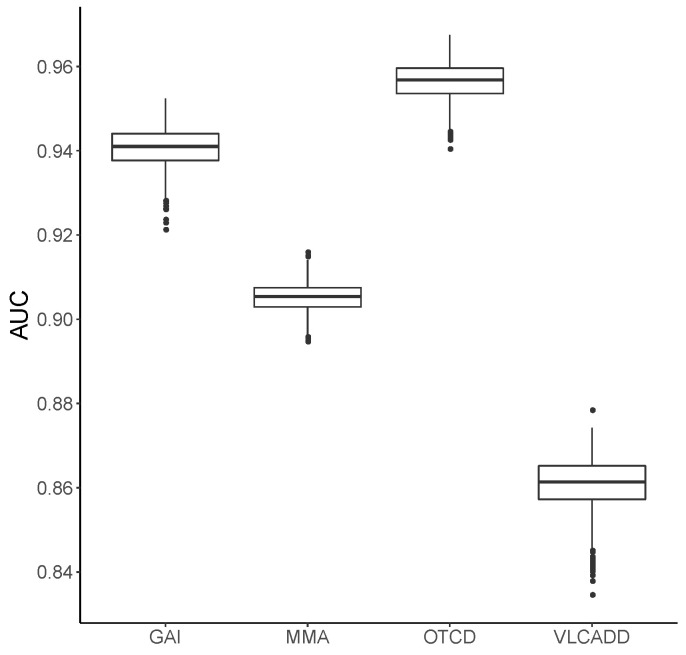
Assessing the performance of RF using cross validation. A 10-fold cross validation (1000 repeats) of the RF model was performed for each disorder to classify each screen positive as either a true or false positive. Only RF scores from testing samples were used to plot the ROC curve and to calculate the AUC. The small variation in AUC values without extreme outlier cases for each disorder demonstrates the overall stability of our RF model.

**Figure 3:**
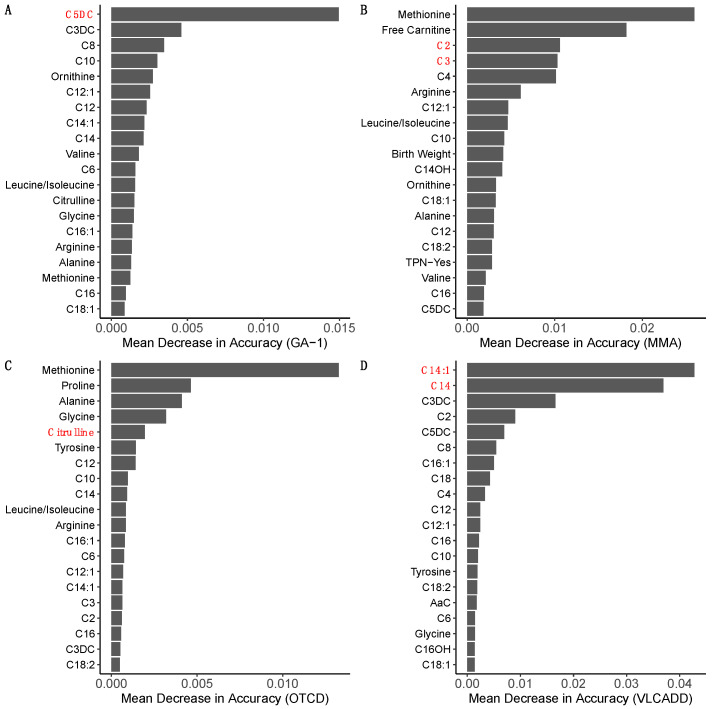
The contribution of individual metabolic analytes in the RF model. The mean decrease in accuracy (MDA) was used to rank the relative importance of individual MS/MS analytes and clinical variables for metabolic pattern recognition in the RF model. Only the 20 top-ranked analytes and variables for each disease are shown: (**A**) GA-1; (**B**) MMA; (**C**) OTCD; (**D**) VLCADD, with the primary markers labeled in red. Abbreviation: Infants with TPN (TPN–Yes); Age at blood collection (AaC).

The LOOCV approach is similar in concept to the analysis of individual screen-positive cases in NBS. However, there is no sampling difference for each repeat in LOOCV and only the final LOOCV error estimate on the testing set is reported [18]. Thus, for each disorder only one AUC is generated without an estimate of variation. To rigorously assess the stability of the RF method, we performed a 10-fold cross validation that was repeated 1000 times for each disorder. For each disorder, all screen-positive cases were divided into ten sample groups with an equal proportion of true and false positives in each group. For example, the 605 MMA screen-positive cases were divided into ten groups each containing approximately 10 true positives and 50 false positives. At each validation step, nine sample groups were combined for training, and one group of blinded samples was used for testing. In result of this 10-fold cross validation, each of the 605 MMA samples received one RF score. Only RF scores from testing cases (and not from training) were used to plot the ROC curve and calculate the AUC. This process was repeated 1000 times for each disorder in order to assess the variation in AUC values (Figure 2). For each disorder, the median number of false positives predicted across the 1000 repeats was based on the sensitivity level of detecting this disorder in the California NBS program. Finally, the mean decrease in accuracy (MDA) was used to measure the contribution of individual metabolic analytes in the RF model (Figure 3). Based on the high correlation between some of the metabolic analytes (Pearson correlation coefficient > 0.9), MDA was selected instead of the alternative approach using mean decrease in Gini (MDG) index [19].

### 2.4. Web-Based RF Tool and Statistical Analysis

Open-source web-based software was developed for the analysis and interpretation of MS/MS data from newborn screening (https://rusptools.shinyapps.io/RandomForest). The new online tool incorporates our RF model for the four studied diseases and was developed with the R shiny package [20], which has been used to build user-friendly interactive web apps with R. The tool’s graphical user interface (GUI) was designed to streamline the process of NBS data reanalysis and to facilitate deployability in the NBS laboratory. A cutoff value was required to separate true- and false-positive cases. The estimated sensitivity for detecting true positives was calculated as the median of sensitivity from our 10-fold CV (1000 repeats). The default sensitivity cutoff in the software corresponds to the current sensitivity of detecting each disorder in the California NBS program. Users can also customize the cutoff value. A cutoff based on high sensitivity indicates a low RF score and low specificity. Detailed description of the input data format, output results, and a user guide are available at https://peng-gang.github.io/RUSP_RF_UserGuide/. Statistical analyses, graphs and design of the research and online tool was done in R software 3.6.1 [21] using these R packages: randomForest [22], ggplot2 [23], pROC [24], caret [25] and shiny [20].

## 3. Results

### 3.1. Metabolic Pattern Analysis Using Random Forest

To demonstrate that machine learning could improve discrimination between true- and false-positive cases without compromising sensitivity, we trained a RF classifier on NBS data from screen positives for four metabolic disorders reported by the California NBS program. Without changing the sensitivity of first-tier screening for these disorders, RF reduced the number of false positives by 89% for GA-1, 45% for MMA, 98% for OTCD and by 2% for VLCADD (Table 1). Accordingly, the positive predictive value (PPV = TP/TP + FP) was significantly improved for three out of the four disorders with a 6.2-fold increase to 22.3% for GA-1, a 0.6-fold increase to 26.4% for MMA and a 16.7-fold increase to 62.1% for OTCD. The performance of RF for the four disorders ranged from an AUC of 0.80 to 1.00 (95% CI) (Figure 1). The ROC curve shows the relationship between sensitivity and specificity in the RF model. Users can choose any point on the ROC curve to select the desired sensitivity for disease screening, while the vertical line through that point corresponds to the specificity of the RF model for detecting the disease, based on the selected sensitivity. To further investigate the potential variability in performance in our RF model, we performed a 10-fold cross validation with 1000 repeats for each disorder. This design maximized the sampling differences during cross validation and revealed only small variations in the AUC for each disorder without extreme outlier cases (Figure 2), which indicated the overall stability of the RF model.

### 3.2. Ranking of Metabolic Analytes

The MDA index was used to identify the individual contribution of specific MS/MS analytes and covariates in our RF model. The 20 top-ranked analytes and variables for each disorder are shown in Figure 3. Notably, each of the primary markers used to detect the four diseases in the California NBS program was among the five top-ranked analytes for each disorder. Two of the top-ranked analytes are part of informative ratios for GA-1 (C8) and MMA (methionine) [26,27], while several other analytes were found to be related to these disorders based on a literature search (Table 3). Except for birth weight and TPN ranked at 10 and 17 for MMA, respectively, none of the clinical variables were among the 20 top-ranked RF features.

### 3.3. Comparison of CLIR and Random Forest

The performance of RF and CLIR postanalytical tools was compared using MS/MS data for GA-1 screen-positive newborns. To preclude any bias in this comparison, 366 of the 1344 false-positive cases in our cohort (Table 1) were removed from this analysis based on the “Tool Runner” function in CLIR. The remaining cohort of 1026 GA-1 screen positives (48 TP and 978 FP) was analyzed with each method. In CLIR, analysis was performed separately for derivatized (407 FP and 25 TP) and underivatized (571 FP and 23 TP) data, while RF analysis was done for all 1026 GA-1 screen positives combined. In result, the number of GA-1 false-positive cases were reduced using CLIR by 93.1% (four false negatives), and using RF by 94.6% (five false negatives) (Table 4). Adjusting the RF score cutoff from 0.12 (default cutoff based on 10-fold cross validation) to 0.086 reduced the number of GA-1 false positives by 92.6% (four false negatives).

### 3.4. Web-Based RF Tool

**Figure 4:**
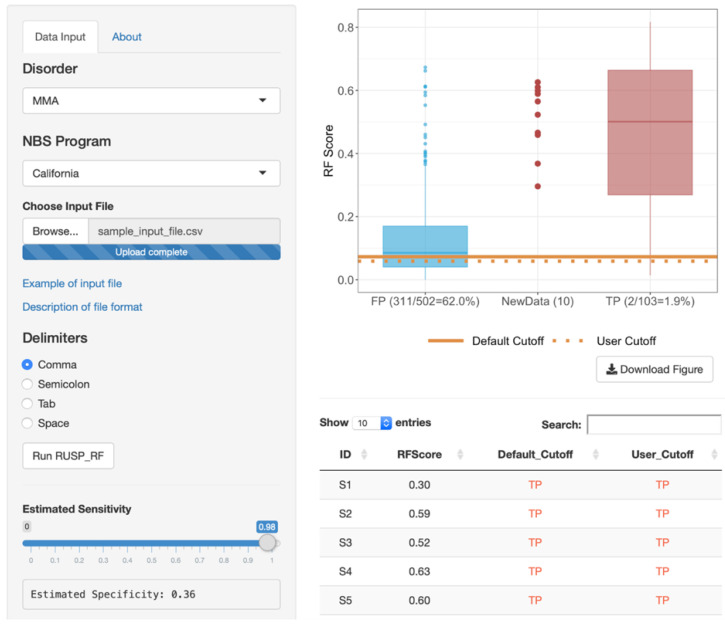
Graphical user interface (GUI) of the web-based Random Forest (RF) tool for NBS data analysis. The menu panel on the left was designed to upload data and select parameters, while the panel on the right shows the results from RF-based analysis of the metabolic input data. Users can select a cutoff value or use the default cutoff, which was calculated for each disorder based on the median sensitivity of 1000 repeats in the 10-fold cross validation (Figure 2).

The new software tool is available at https://rusptools.shinyapps.io/RandomForest/ and the GUI is shown in Figure 4. The RUSPtools user guide is also available under Supplementary Material. The workflow starts with selecting a “disorder” and a “NBS program” reporting the data, which in this example is MMA and California, respectively. Users then upload a MS/MS sample data file (i.e., sample_input_file.csv in the website) and click “Run RUSP_RF.” Output results containing two boxplots and a table are shown on the right panel, which is being computed in less than 30 seconds depending on input file size and server connection. An error message is provided for an incompatible user file format. The boxplots show the distribution of the RF score for each sample in the groups of false positives (blue boxplot, left) and true positives (red boxplot, right). The RF score of user-uploaded samples are shown in between the two boxplots. The table in Figure 4 lists the corresponding RF predictions (TP or FP) for each sample based on a suggested RF cutoff value. This default sensitivity cutoff was set to be the same as the sensitivity in the state NBS program for each disorder. Users can also customize a cutoff value in the left panel in the online tool.

## 4. Discussion

Although MS/MS screening identifies most infants with a metabolic disorder on the RUSP, it also creates a high number of false positives that require additional confirmatory testing of all screen-positive cases. At present, NBS relies on the detection of abnormal levels of only one or a few disease-specific markers and their ratios. We recently showed improved separation of true and false-positive cases through Random Forest-based analysis of all analytes on the MS/MS screening panel [15]. Here we expanded this RF-based approach for analysis of four metabolic disorders (GA-1, MMA, OTCD and VLCADD), each of which is compromised by high false-positive rates and diagnostic delays following a positive newborn screen. Without changing the sensitivity for detecting these disorders in screening, RF was able to reduce the number of false positives by 89% for GA-1, 45% for MMA, 98% for OTCD and by 2% for VLCADD (Figure 1). By reducing false positives in first-tier screening, this RF-based second-tier approach increased the PPV, and in particular for detecting GA-1 (from 3% to 22%) and OTCD (3% to 62%) (Table 1). These results support our previous findings of improved performance using RF-based analysis of the entire newborn metabolic profile [15].

Metabolic analytes with a large mean decrease in accuracy (MDA) in the RF model are more important for classification of disease status. MDA was used to identify the top-ranked MS/MS analytes and clinical variables for each disorder (Figure 3). All primary MS/MS markers currently in use for identifying screen positives for the four disorders in the California NBS program were among the five top-ranked analytes (Table 3). RF also identified several secondary analytes that are part of important analyte ratios with primary analytes for GA-1 (C5DC/C8), MMA (C3/Methionine) and VLCADD (C14:1/C2) [26,27,28,34,35]. Methionine, which was top ranked by MDA analysis for MMA, has been associated with differences in MMA phenotypic subgroups, with lower levels in patients with remethylation defects (CblC, D or F) compared to mutase deficiency (mut^0/−^) [15,30]. Notably, methionine was also the top-ranked analyte in the RF model for reducing OTCD false positives (Figure 3). The methionine/citrulline ratio was identified as an OTCD screening marker [30]. Similar in concept to separating MMA subgroups, these results suggest that methionine could be associated with OTCD phenotypic subgroups. However, abnormal levels of multiple serum amino acids such as methionine, proline, alanine and glycine could also be a sign of generalized liver damage seen in OTCD patients [29]. In contrast to the other three diseases, there was only a very small reduction in false-positive cases for VLCADD, which indicates the need for discovery of novel screening markers and molecular confirmatory testing to identify VLCADD carriers who could mistakenly be classified as false positives [37]. In comparison, a retrospective study using R4S tools showed that sequential postanalytical analysis could have reduced follow-up testing in 25.8% of VLCADD cases [38].

Random Forest incorporates information from all metabolic analytes and clinical variables collected at birth. Analytes and variables with lower association to a particular disorder would be assigned a smaller weight in RF and downranked in the MDA analysis. By including clinical variables in RF, the metabolic analytes can be adjusted in relation to the variable. For example, if an analyte level was higher in males than in females, the cutoff value for this analyte would be automatically adjusted higher for a male compared to a female. The inclusion of additional important analyte ratios could further improve RF performance. Because it may be difficult to simultaneously adjust the levels of many analytes for multiple interacting variables, RF provides a new solution for this problem by directly integrating all the information from screening into a single RF score. A single RF score could improve prediction of metabolic disease status, and particularly as the amount of NBS data and the consequent challenges of analyzing these data increases in the future.

To further evaluate the performance of RF, a comparison to CLIR postanalytical tools was performed. Using MS/MS data for GA-1 screen-positive cases, the performances of CLIR and RF were found to be similar for predicting false positives (Table 4). Based on the default RF score cutoff, RF predicted 14 fewer false positives and one more false negative compared to CLIR. Lowering the RF cutoff to reach the same sensitivity as CLIR resulted in four false negatives (same as CLIR) and 72 false positives (five more than CLIR). Notably, CLIR incorporates several millions of normal screening test results and profiles of screen-positive cases from NBS programs across the US and worldwide. The RF tool in comparison is currently limited to only the data from this study in one state (California NBS program) and four diseases. Similar in concept to CLIR, additional NBS data could be readily incorporated and further improve RF-based predictions. RF and CLIR utilize different methodologies with different advantages for reducing FP screens. When comparing results between CLIR and RF for detecting GA-1 false-positive cases, we found that 40 infants were categorized as TP by CLIR and as FP by RF, while an additional 26 infants were categorized as TP by RF and as FP by CLIR, respectively. Results from the two tools could be integrated using ensemble methods to achieve better predictive performance than could be obtained from each single method alone.

We note that data for metabolic analytes and clinical variables may be collected differently across NBS programs. Age at blood collection, for example, is an important covariate for metabolite levels [39], and some states may collect blood spots earlier than 24 hours. Age at collection was included in the RF model to adjust for its effect on marker levels and to make the algorithm applicable to other NBS programs. However, there may be other distinguishing factors that limit the application of this RF model (built using CA NBS data) for these programs. To address this problem, we could either collect data from different NBS programs and make adjustment in the RF tool (e.g., batch effect correction), or develop different RF models that are tailored to specific needs of each program.

To facilitate broader application of RF in second-tier analysis and interpretation, we established a novel web-based software (https://rusptools.shinyapps.io/RandomForest/). This RF tool could be of primary interest to NBS reference laboratories for evaluating MS/MS data from screen-positive cases. Analysis of individual NBS data and prediction of false-positive screens can be obtained within minutes, given the RF model has been established for that particular disease. However, RF-based predictions should always be considered in conjunction with established second-tier confirmatory analysis using biochemical and DNA testing of all screen-positive cases. Ideally, such combined analysis should be performed more rapidly to reduce the number of “false alarms” and positive callouts before parent contact. This is particularly important for inborn metabolic disorders that can present in the first weeks of life and require fast turnaround time of NBS results. The new open-source software creates a low barrier for entry that enables users to rapidly analyze case data, and in turn help improve the RF algorithm for newborn screening.

## Figures and Tables

**Table 1:** Number of patients, false positives and PPV of first and second-tier testing (newborn screening (NBS), glutaric acidemia type 1 (GA-1), methylmalonic acidemia (MMA), ornithine transcarbamylase deficiency (OTCD), or very long-chain acyl-CoA dehydrogenase deficiency (VLCADD)).

Disorder	Confirmed Positive	First-Tier NBS		Second-Tier Analysis (RF, This Study)	
		False Positives	PPV	False Positives *	PPV
GA-1	48	1344	3.10%	150	22.30%
MMA	103	502	16.40%	276	26.40%
OTCD	24	496	3.50%	11	62.10%
VLCADD	60	200	23.10%	196	23.40%

* Median of false positives from 1000 repeats of 10-fold CV.

**Table 2:** Participant and Subgroup Demographics for four disorders.

	GA-1	MMA	OTCD	VLCADD	Control *
Gestational Age, week					
<37	340 (24.4%)	175 (28.9%)	181 (34.8%)	42 (16.2%)	5490 (5.5%)
37–41	1005 (72.2%)	412 (68.1%)	325 (62.5%)	206 (79.2%)	93,603 (94.0%)
>41	47 (3.4%)	18 (3.0%)	14 (2.7%)	12 (4.6%)	444 (0.4%)
Birth Weight, g					
<2500	279 (20.0%)	173 (28.6%)	130 (25.0%)	26 (10.0%)	4045 (4.1%)
2500–4000	1025 (73.6%)	381 (63.0%)	354 (68.1%)	223 (85.8%)	87,268 (87.7%)
>4000	88 (6.3%)	51 (8.4%)	36 (6.9%)	11 (4.2%)	8224 (8.3%)
Sex					
Male	845 (60.7%)	321 (53.1%)	325 (62.5%)	165 (63.5%)	51,352 (51.6%)
Female	542 (38.9%)	281 (46.4%)	194 (37.3%)	93 (35.8%)	47,882 (48.1%)
Unknown	5 (0.4%)	3 (0.5%)	1 (0.2%)	2 (0.8%)	303 (0.3%)
Race/Ethnicity					
Asian	136 (9.8%)	63 (10.4%)	33 (6.3%)	40 (15.4%)	14275 (14.3%)
Black	212 (15.2%)	25 (4.1%)	50 (9.6%)	15 (5.8%)	6630 (6.7%)
Hispanic	444 (31.9%)	407 (67.3%)	224 (43.1%)	94 (36.2%)	49,400 (49.6%)
White	554 (39.8%)	92 (15.2%)	197 (37.9%)	102 (39.2%)	26341 (26.5%)
Other/Unknown	46 (3.3%)	18 (3.0%)	16 (3.1%)	9 (3.5%)	2891 (2.9%)
Age at Blood Collection, hour					
<12	246 (17.7%)	142 (23.5%)	45 (8.7%)	47 (18.1%)	21,564 (21.7%)
12–24	877 (63.0%)	319 (52.7%)	259 (49.8%)	183 (70.4%)	71,396 (71.7%)
>24	269 (19.3%)	144 (23.8%)	216 (41.5%)	30 (11.5%)	6577 (6.6%)
Total Parenteral Nutrition					
No	1178 (84.6%)	393 (65.0%)	453 (87.1%)	248 (95.4%)	97,269 (97.7%)
Yes	146 (10.5%)	187 (30.9%)	57 (11.0%)	3 (1.2%)	998 (1.0%)
Unknown	68 (4.9%)	25 (4.1%)	10 (1.9%)	9 (3.5%)	1270 (1.3%)

* The number and percentage were calculated from 99,537 singleton screen-negative newborns randomly selected from the California NBS program between 2013 to 2015.

**Table 3:** The five top-ranked analytes identified by Random Forest for each disease.

MDA Ranking	GA-1	MMA	OTCD	VLCADD
1	C5DC ^a^	Methionine ^b^ [28]	Methionine ^b^ [29,30]	C14:1 ^a^
2	C3DC	Free Carnitine [31]	Proline ^b^ [29,32,33]	C14 ^a^
3	C8 ^b^ [28]	C2 ^a^	Alanine [29]	C3DC
4	C10 [34]	C3 ^a^	Glycine [29]	C2 ^b^ [35]
5	Ornithine [36]	C4	Citrulline ^a^	C5DC

^a^ Metabolic analytes used as the primary marker for each disorder in California NBS program. ^b^ Metabolic analytes used as part of informative marker ratios included C5DC/C8 for GA-1, C3/Met for MMA (CblC, D or F), methionine/citrulline and proline/citrulline for OTCD, and C14:1/C2 for VLCADD. Additional references provide support for analytes identified in this study.

**Table 4:** Comparison of CLIR and Random Forest for GA-1 screen positives (cleaned data) (true positive (TP) and false positive (FP)).

	Predicted by Algorithm	NBS Results (Truth)	
		TP	FP
CLIR	TP	44	67
	FP	4	911
Random Forest	TP	43	53
	FP	5	925

## Supplementary Materials

Supplementary materials can be found at https://www.mdpi.com/2409-515X/6/1/16/s1.

## References

[1] Kwon C., Farrell P.M. (2000). The magnitude and challenge of false-positive newborn screening test results. Arch. Pediatr. Adolesc. Med..

[2] Marquardt G., Currier R., McHugh D.M.S., Gavrilov D., Magera M.J., Matern D., Oglesbee D., Raymond K., Rinaldo P., Smith E.H. (2012). Enhanced interpretation of newborn screening results without analyte cutoff values. Genet. Med..

[3] Tortorelli S., Eckerman J.S., Orsini J.J., Stevens C., Hart J., Hall P.L., Alexander J.J., Gavrilov D., Oglesbee D., Raymond K. (2018). Moonlighting newborn screening markers: The incidental discovery of a second-tier test for Pompe disease. Genet. Med..

[4] Minter Baerg M.M., Stoway S.D., Hart J., Mott L., Peck D.S., Nett S.L., Eckerman J.S., Lacey J.M., Turgeon C.T., Gavrilov D. (2018). Precision newborn screening for lysosomal disorders. Genet. Med..

[5] Hall P.L., Marquardt G., McHugh D.M., Currier R.J., Tang T., Stoway S.D., Rinaldo P. (2014). Postanalytical tools improve performance of newborn screening by tandem mass spectrometry. Genet. Med..

[6] Morkrid L., Rowe A.D., Elgstoen K.B., Olesen J.H., Ruijter G., Hall P.L., Tortorelli S., Schulze A., Kyriakopoulou L., Wamelink M.M. (2015). Continuous age- and sex-adjusted reference intervals of urinary markers for cerebral creatine deficiency syndromes: A novel approach to the definition of reference intervals. Clin. Chem..

[7] Baumgartner C., Bohm C., Baumgartner D., Marini G., Weinberger K., Olgemöller B., Liebl B., Roscher A.A. (2004). Supervised machine learning techniques for the classification of metabolic disorders in newborns. Bioinformatics.

[8] Chen W.H., Hsieh S.L., Hsu K.P., Chen H.P., Su X.Y., Tseng Y.J., Chien Y.H., Hwu W.L., Lai F. (2013). Web-based newborn screening system for metabolic diseases: Machine learning versus clinicians. J. Med. Internet Res..

[9] Ho T.K. Random decision forests. Paper Presented at: Proceedings of the 3rd International Conference on Document Analysis and Recognition.

[10] Breiman L. (2001). Random forests. Mach. Learn..

[11] Chen X., Ishwaran H. (2012). Random forests for genomic data analysis. Genomics.

[12] Wu B., Abbott T., Fishman D., McMurray W., Mor G., Stone K., Ward D., Williams K., Zhao H. (2003). Comparison of statistical methods for classification of ovarian cancer using mass spectrometry data. Bioinformatics.

[13] Melo C., Navarro L.C., de Oliveira D.N., Guerreiro T.M., Lima E.O., Delafiori J., Dabaja M.Z., Ribeiro M.D.S., de Menezes M., Rodrigues R.G.M. (2018). A Machine Learning Application Based in Random Forest for Integrating Mass Spectrometry-Based Metabolomic Data: A Simple Screening Method for Patients With Zika Virus. Front. Bioeng Biotechnol..

[14] Kopp B.T., Joseloff E., Goetz D., Ingram B., Heltshe S.L., Leung D.H., Ramsey B.W., McCoy K., Borowitz D. (2019). Urinary metabolomics reveals unique metabolic signatures in infants with cystic fibrosis. J. Cyst. Fibros..

[15] Peng G., Shen P., Gandotra N., Le A., Fung E., Jelliffe-Pawlowski L., Davis R.W., Enns G.M., Zhao H., Cowan T.M. (2019). Combining newborn metabolic and DNA analysis for second-tier testing of methylmalonic acidemia. Genet. Med..

[16] American College of Medical Genetics Newborn Screening Expert Group (2006). Newborn screening: Toward a uniform screening panel and system—Executive summary. Pediatrics.

[17] Oshiro T.M., Perez P.S., Baranauskas. J.A., Perner P. (2012). How Many Trees in a Random Forest?. Machine Learning and Data Mining in Pattern Recognition.

[18] Varma S., Simon R. (2006). Bias in error estimation when using cross-validation for model selection. BMC Bioinform..

[19] Nicodemus K.K. (2011). Letter to the editor: On the stability and ranking of predictors from random forest variable importance measures. Brief. Bioinform..

[20] Shiny: Web Application Framework for R. https://shiny.rstudio.com.

[21] R: A Language and Environment for Statistical Computing. https://www.r-project.org.

[22] Liaw A., Wiener M. (2002). Classification and regression by randomForest. R. News..

[23] Wickham H. (2016). Ggplot2: Elegant Graphics for Data Analysis.

[24] Robin X., Turck N., Hainard A., Tiberti N., Lisacek F., Sanchez J.-C.,  Müller M. (2011). pROC: An open-source package for R and S+ to analyze and compare ROC curves. BMC Bioinform..

[25] Kuhn M. (2008). Building predictive models in R using the caret package. J. Stat. Softw..

[26] Zytkovicz T.H., Fitzgerald E.F., Marsden D., Larson C.A., Shih V.E., Johnson D.M., Strauss A.W., Comeau A.M., Eaton R.B., Grady G.F. (2001). Tandem mass spectrometric analysis for amino, organic, and fatty acid disorders in newborn dried blood spots: A two-year summary from the New England Newborn Screening Program. Clin. Chem..

[27] Weisfeld-Adams J.D., Morrissey M.A., Kirmse B.M., Salveson B.R., Wasserstein M.P., McGuire P.J., Sunny S., Cohen-Pfeffer J.L., Yu C., Caggana M. (2010). Newborn screening and early biochemical follow-up in combined methylmalonic aciduria and homocystinuria, cblC type, and utility of methionine as a secondary screening analyte. Mol. Genet. Metab..

[28] Rinaldo P., Whitley R.J., Rhead W.J., Hannon W.H. (2009). Evidence-Based Rationale for Expanded Newborn Screening. N. Engl. J. Med..

[29] McClead R.E.J., Rozen R., Fox J., Rosenberg L., Menke J., Bickers R., Morrow G. (1986). Clinical application of DNA analysis in a family with OTC deficiency. Am. J. Med. Genet..

[30] McHugh D., Cameron C.A., Abdenur J.E., Abdulrahman M., Adair O., Al Nuaimi S.A., Ahlman H., Allen J.J., Antonozzi I., Archer S. (2011). Clinical validation of cutoff target ranges in newborn screening of metabolic disorders by tandem mass spectrometry: A worldwide collaborative project. Genet. Med..

[31] Di Donato S., Rimoldi M., Garavaglia B., Uziel G. (1984). Propionylcarnitine excretion in propionic and methylmalonic acidurias: A cause of carnitine deficiency. Clin. Chim. Acta.

[32] Bisanzi S., Morrone A., Donati M.A., Pasquini E., Spada M., Strisciuglio P., Parenti G., Parini R., Papadia F., Zammarchi E. (2002). Genetic analysis in nine unrelated Italian patients affected by OTC deficiency: Detection of novel mutations in the OTC gene. Mol. Genet. Metab..

[33] De Sain-van der Velden M.G., Rinaldo P., Elvers B., Henderson M., Walter J.H., Prinsen B.H., Verhoeven-Duif N.M., de Koning T.J., van Hasselt P. (2012). The Proline/Citrulline Ratio as a Biomarker for OAT Deficiency in Early Infancy. JIMD Rep..

[34] Hennermann J.B., Roloff S., Gellermann J., Gruters A., Klein J. (2009). False-positive newborn screening mimicking glutaric aciduria type I in infants with renal insufficiency. J. Inherited Metab. Dis..

[35] Diekman E., de Sain-van der Velden M., Waterham H., Kluijtmans L., Schielen P., van Veen E.B., Ferdinandusse S., Wijburg F., Visser G. (2016). The Newborn Screening Paradox: Sensitivity vs. Overdiagnosis in VLCAD Deficiency. JIMD Rep..

[36] Kolker S., Boy S.P., Heringer J., Müller E., Maier E.M., Ensenauer R., Mühlhausen C., Schlune A., Greenberg C.R., Koeller D.M. (2012). Complementary dietary treatment using lysine-free, arginine-fortified amino acid supplements in glutaric aciduria type I—A decade of experience. Mol. Genet. Metab..

[37] Atkins A.E., Tarini B.A., Phillips E.K., Calhoun A. (2019). Misclassification of VLCAD carriers due to variable confirmatory testing after a positive NBS result. J. Community Genet..

[38] Merritt J.L., Vedal S., Abdenur J.E., Au S.M., Barshop B.A., Feuchtbaum L., Harding C.O., Hermerath C., Lorey F., Sesser D.E. (2014). Infants suspected to have very-long chain acyl-CoA dehydrogenase deficiency from newborn screening. Mol. Genet. Metab..

[39] Vernooij-van Langen A.M., Loeber J.G., Elvers B., Triepels R.H., Roefs J., Gille J.J., Reijntjens S., Dompeling E., Dankert-Roelse J.E. (2013). The influence of sex, gestational age, birth weight, blood transfusion, and timing of the heel prick on the pancreatitis-associated protein concentration in newborn screening for cystic fibrosis. J. Inherited Metab. Dis..

