# Supplementary material for: Reducing False-Positive Results in Newborn Screening Using Machine Learning
Source: Int J Neonatal Screen. 2020 Mar 3;6(1):16. doi: 10.3390/ijns6010016 (PMC7080200; doi:10.3390/ijns6010016)
Supplement: Supplementary file 1 [file IJNS-06-00016-s001.pdf]

User Guide for Newborn Screening with Random Forest

To facilitate broader application of Random Forest in second-tier analysis and interpretation, a web-based software was established (https://rusptools.shinyapps.io/RandomForest/). The new RF-tool is based on the Random Forest method[1] and aims to reduce the false positive rate in NBS by incorporating data from all metabolic analytes. The workflow of the new RF-tool is shown in **Figure S1**. Users can select a disorder and a State NBS program in which the data were generated, and then chose an input data file that is uploaded to the server. A detailed description of input data file formats is available below. After completed data upload, users click Run RUSP_RF to start the analysis. Results are shown as both a Figure and Table on the right side of the window.

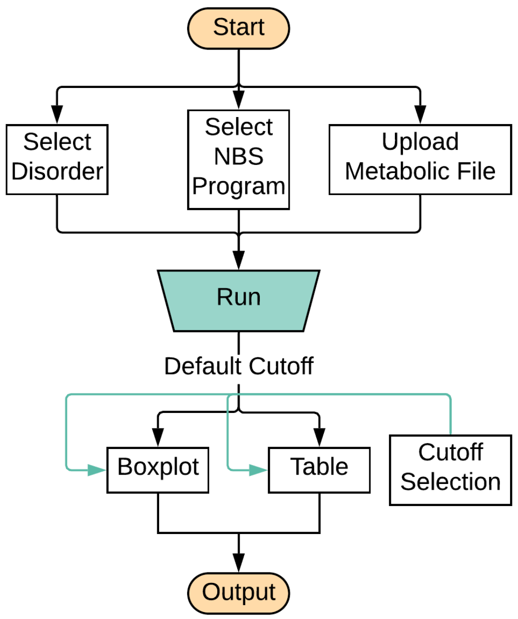

Figure S1. Workflow of the Random Forest online tool.

Instructions

1. Click **Disorder** to select a specific disease from the list.
2. Click **NBS Program** to switch between different NBS programs (currently CA data only).
3. Click **Browse** to choose an input data file (1 or multiple samples) from your local device.
4. Select a **Delimiter** in your input data file and click **RUSP_RF** to start analysis.
5. Inspect the **Results** of the analysis in the figure and table on the right side.
6. A default sensitivity cutoff for the **RF score** is provided to quickly identify true- and false positive cases among the input samples. The sensitivity cutoff is **customizable** using the slider bar on the left-side panel.
7. **Individual samples** listed in the results table can be clicked and visualized in the figure. (Click the table row again to cancel the selection.)
8. **Search table** by sample ID, cutoff, TP or FP to customize the results table.
9. Click **Download** Figure or Table download the analysis results to your local device.
10. **Note:** Results from the analysis for a particular disease will be cleared once a user navigates to a different disease. A note is displayed to prevent this from happening. Please download results for a disease analysis before navigating to a different disease.

Input Data Format

The RF-tool requires 3 data inputs: Disorder, NBS Program, and Metabolic analyte data.

Disorder: The disorder is specified by selecting the disorder name on the sidebar. There are four disorders included in the current application: GA-1 (Glutaric Acidemia Type I), MMA (Methylmalonic Acidemia), OTCD (Ornithine transcarbamylase deficiency), and VLCADD (Very Long-chain Acyl-CoA Dehydrogenase Deficiency).

NBS Program: The NBS program indicates the state in which the NBS data were generated (e.g., the birthplace of the newborn). The current RF-tool was validated based on data provided by the California NBS program. Considering state-by-state difference in newborn screening for metabolic disorders (e.g., composition of MS/MS panels, cutoff values), the current RF-model may not be applicable to NBS data from other states. However, similar to the current model, a new RF model could be readily established based on data from another NBS programs. We welcome inquiries for establishing a new RF model for data from other NBS programs.

Metabolic data: The NBS input data can be uploaded in a variety of file formats including comma separated values (csv), or plain text format with its delimiters being one of the following: semicolon, tab, or space. The first row of the file contains the header information for each column with analyte names. Each row after the header row contains the sample data (e.g., one row per sample). The 39 marker columns can be in any order as long as the total number of columns is 39. A description and format of each column are described shown in **Table S1**, and a list of acceptable header field names is shown in **Table S2**.

Table 1. Description of data input file format.

**Column**	**1**	**2 - 40**
Headers	id	Original marker name or its abbreviation
Example Headers	ID, sample	Citrulline, cit, C18:1, c181, C5-OH, C5OH
Values	unique sample id (string)	Individual analyte level (numeric)
Example Values	S01, sample_001	0.2, 0.11421383

Table 2. Header field names accepted by the online RF-tool.

Headers	Possible alternative naming
id	ID, SAMPLE, SAMPLE IDENTIFIER, SAMPLE ID, SID, IDENTIFIER
GLY	GLY, GLYCINE
ALA	ALA, ALANINE
PRO	PRO, PROLINE
VAL	VAL, VALINE
OXP	OXP, 5-OXOPROLINE, pyroglutamic acid, GLP
XLE	XLE, LEU-ILE, LEU+ILE, LEUCINE+ISOLEUCINE, LEUCINE AND ISOLEUCINE, LEUCINE OR ISOLEUCINE
ORN	ORN, ORNITHINE
MET	MET, METHIONINE
ARG	ARG, ARGININE
CIT	CIT, CITRULLINE
PHE	PHE, PHENYLALANINE
TYR	TYR, TYROSINE
C0	C0, FREE CARNITINE, FC
C2	C02, C2
C3	C03, C3
C4	C04, C4
C5:1	C051, C51, C5:1
C5	C05, C5
C6	C06, C6
C8:1	C081, C8:1, C81
C8	C08, C8
C10:1	C101, C10:1
C10	C10
C12:1	C121, C12:1
C12	C12
C14:1	C141, C14:1
C14	C14
C14OH	C14OH, C14-OH
C16:1	C161, C16:1
C16	C16
C16OH	C16OH, C16-OH
C18:2	C182, C18:2
C18:1	C181, C18:1
C18	C18
C18:1OH	C181OH, C18:1OH, C18:1-OH
C18OH	C18OH, C18-OH
C5OH	C05OH, C5OH, C5-OH
C3DC	C03DC, C3DC, C3-DC
C5DC	C05DC, C5DC, C5-DC

Methods

There are many trees in a random forest (RF)[1] model and each RF tree has one vote in a binary classification model. We defined the RF score as the fraction of true positive votes among all votes. The ROC curve is drawn from the RF score. In our study, a high RF score indicates high probability of a true-positive case, while a low RF score indicates a high probability of a false positive. There is a direct correlation between the RF score and screening sensitivity. In this analysis we decided to use sensitivity as the cutoff for the RF score to separate true- and false positives in the RF model. In order to suggest a default RF score cutoff, we repeated the 10-fold cross validation for 1000 times and defined a RF Score threshold that achieves the same sensitivity as the state NBS program at each repeat. We considered the median RF score across the 1000 repeats that achieved the expected sensitivity of the state NBS program.

Results

The results of the RF tool are shown in the right side of the window with a boxplot on the top and a table on the bottom. The boxplot (**Figure S2**) shows the distribution of the RF scores (y axis for each sample calculated from LOOCV in the false-positive (FP, blue) and true-positive (TP, red) groups. The RF scores for the user's input sample data is shown in the center (e.g., "New Data"). Users can select a cutoff for screening sensitivity that directly correlates with the RF score. In order to help users to make this decision, we have suggested a default RF score cutoff in the RF tool (see Methods section). Users can also select the cutoff based on their input data. The suggested RF score cutoff is shown as solid line, while the user-selected cutoff is shown as a dashed line. When changing the cutoff for sensitivity, an estimation of specificity is shown for both the TP and FP groups. A low RF score corresponds to high sensitivity and low specificity. In **Figure S2**, the number for false-positives is "311/502=62.0%" and the number for true-positives is "2/103 = 1.9%". Thus, based on this user's selected cutoff, 311 of the 502 false-positives from primary NBS remain were classified as false-positives, while only 2 of the 103 true-positive cases remained as false-negatives (2 fewer false-negatives than in primary NBS).

Additional notes

The shiny web-tool will not track or record user's input data or results. Please send any comments or questions by clicking 'Report issues to the developers'.

References

1. Breiman, Leo. 2001. "Random Forests." *Machine Learning* 45 (1). Springer: 5–32.

Figure S2. Analysis of newborn metabolic screening data using the RF-tool. Shown are the results from RF-based analysis of metabolic input data (New data, 1 dot per sample) in comparison to groups of false-positive cases (FP, blue boxplot) and true-positive cases (TP, red boxplot) with the same disorders. The default RF score cutoff (solid line) is based on the median sensitivity for detecting true-positive case for this disorder in the 10-fold cross validation. The user-defined cutoff (dashed line) can be adjusted based on desired number of true-positive (sensitivity) and false-positive cases in the user's data set. The table includes four columns with sample id's, RF score, and the results from the default and the user-selected cutoffs.

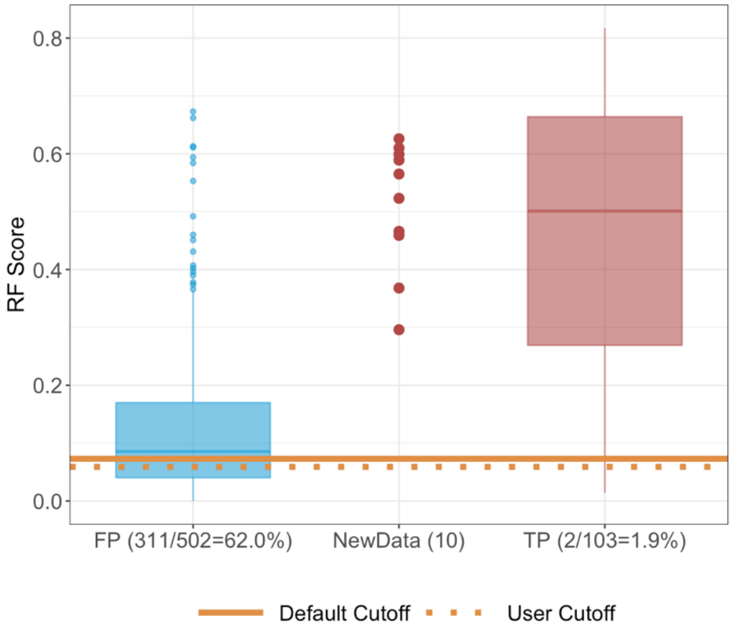

ID	RFScore	Default_Cutoff	User_Cutoff
S1	0.30	TP	TP
S2	0.59	TP	TP
S3	0.52	TP	TP
S4	0.63	TP	TP
S5	0.60	TP	TP
S6	0.61	TP	TP
S7	0.56	TP	TP
S8	0.47	TP	TP
S9	0.37	TP	TP
S10	0.46	TP	TP
